# Mass Testing for SARS-CoV-2 in 16 Prisons and Jails — Six Jurisdictions, United States, April–May 2020

**DOI:** 10.15585/mmwr.mm6933a3

**Published:** 2020-08-21

**Authors:** Liesl M. Hagan, Samantha P. Williams, Anne C. Spaulding, Robin L. Toblin, Jessica Figlenski, Jeanne Ocampo, Tara Ross, Heidi Bauer, Justine Hutchinson, Kimberley D. Lucas, Matthew Zahn, Chun Chiang, Timothy Collins, Alexis Burakoff, Juli Bettridge, Ginger Stringer, Randolph Maul, Kristen Waters, Courtney Dewart, Jennifer Clayton, Sietske de Fijter, Radha Sadacharan, Linda Garcia, Naomi Lockett, Kirstin Short, Laxman Sunder, Senad Handanagic

**Affiliations:** ^1^CDC; ^2^Emory University Rollins School of Public Health, Atlanta, Georgia; ^3^Morehouse School of Medicine, Atlanta, Georgia; ^4^Federal Bureau of Prisons, Washington, DC; ^5^California Correctional Health Care Services; ^6^Orange County Health Care Agency, Santa Ana, California; ^7^Colorado Department of Public Health and Environment; ^8^Colorado Department of Corrections; ^9^Ohio Department of Health; ^10^Epidemic Intelligence Service, CDC; ^11^Ohio Department of Rehabilitation and Correction; ^12^Brown University Department of Family Medicine, Providence, Rhode Island; ^13^Harris County Sheriff’s Office, Houston, Texas; ^14^Houston Health Department, Houston, Texas.

Preventing coronavirus disease 2019 (COVID-19) in correctional and detention facilities* can be challenging because of population-dense housing, varied access to hygiene facilities and supplies, and limited space for isolation and quarantine (*1*). Incarcerated and detained populations have a high prevalence of chronic diseases, increasing their risk for severe COVID-19–associated illness and making early detection critical (*2*,*3*). Correctional and detention facilities are not closed systems; SARS-CoV-2, the virus that causes COVID-19, can be transmitted to and from the surrounding community through staff member and visitor movements as well as entry, transfer, and release of incarcerated and detained persons (*1*). To better understand SARS-CoV-2 prevalence in these settings, CDC requested data from 15 jurisdictions describing results of mass testing events among incarcerated and detained persons and cases identified through earlier symptom-based testing. Six jurisdictions reported SARS-CoV-2 prevalence of 0%–86.8% (median = 29.3%) from mass testing events in 16 adult facilities. Before mass testing, 15 of the 16 facilities had identified at least one COVID-19 case among incarcerated or detained persons using symptom-based testing, and mass testing increased the total number of known cases from 642 to 8,239. Case surveillance from symptom-based testing has likely underestimated SARS-CoV-2 prevalence in correctional and detention facilities. Broad-based testing can provide a more accurate assessment of prevalence and generate data to help control transmission (*4*).

In May 2020, CDC requested data from 15 jurisdictions (the Federal Bureau of Prisons [BOP], 10 state prison systems, and four city or county jails), describing SARS-CoV-2 mass testing events^†^ and cases identified before mass testing. Jurisdictions were selected based on previous discussions with investigators about mass testing events that had already occurred. Six jurisdictions provided data from 16 adult facilities, including the number of COVID-19 cases identified among incarcerated or detained persons and staff members before mass testing and findings from subsequent mass testing events^§^ among incarcerated or detained persons. Data describing mass testing of staff members were not available. One jurisdiction also provided results of retesting among quarantined close contacts of persons with COVID-19, 7 days after their initial negative test result from mass testing. All jurisdictions provided qualitative information describing testing practices before mass testing, actions taken based on mass testing results, and barriers to future broad-based testing. SARS-CoV-2 prevalence was calculated within each facility and by housing type. The numbers of known cases before and after mass testing were compared. Qualitative data were summarized. All analyses were descriptive; significance testing was not performed. This investigation was reviewed by CDC for human subjects protection and determined to be nonresearch.^¶^

Six of the 15 queried jurisdictions (BOP, three state prison systems, and two county jails) provided aggregate, facility-level data representing 16 adult facilities (11 state prisons, three federal prisons, and two county jails). From the beginning of the COVID-19 pandemic until the date of their respective mass testing events, four facilities limited testing among incarcerated or detained persons to those with symptoms, and 12 also tested close contacts; six facilities tested small numbers of symptomatic staff members, and 10 advised staff members to seek testing from their own health care providers or health department.

All 16 facilities had identified at least one case through symptom-based testing before mass testing was conducted; the first case was identified among staff members in nine facilities, among incarcerated or detained persons in six, and in both groups the same day in one. One facility identified a case only among incarcerated or detained persons (no staff member cases), and one facility identified a case only among staff members. The number of cases identified using symptom-based testing ranged from 0 to 181 (median = 19) among incarcerated or detained persons and 0 to 257 (median = 10) among staff members.

Mass testing in the 16 facilities was conducted during April 11–May 20. The interval between identification of the first symptomatic case and the start of mass testing ranged from 2 to 41 days (median = 25 days). Across facilities, 16,392 incarcerated or detained persons were offered testing, representing 2.3%–99.6% (median = 54.9%) of facilities’ total populations; 7,597 previously unrecognized infections were identified (Table). All 15 facilities that had identified at least one case among incarcerated or detained persons through earlier symptom-based testing identified additional cases through mass testing (range = 8–2,179; median = 374). Mass testing increased total known cases from 642 (range = 2–181, median = 19) before mass testing to 8,239 (range = 10–2,193, median = 403) after mass testing (Figure), representing a 1.5–157-fold increase (median 12.3-fold) in each facility. The single facility that had identified no cases among incarcerated or detained persons before mass testing also found no cases during mass testing; with this facility included, the median fold-increase in total known cases after mass testing decreased slightly to 12.1-fold. In the 16 facilities, SARS-CoV-2 prevalence found during mass testing among incarcerated or detained persons ranged from 0% to 86.8% (median = 29.3%). Testing refusal rates ranged from 0.0% to 17.3% (median = 0.0%) (Table).

**TABLE:** Results of SARS-CoV-2 mass testing events* among incarcerated or detained persons in 16 prisons and jails — six jurisdictions, United States, April–May 2020

Jurisdiction/Facility	No. of days between identification of first case and start of mass testing^†^	Total persons incarcerated or detained in the facility during mass testing^§^	No. (%) offered testing^¶^	No. (%) who declined testing	No. (%) tested	No. with interpretable results	No. (%) testing positive	Type of housing in tested units (open dorm, cells, or both)**
**Federal Bureau of Prisons^††^**								
Prison 1	25	1,534	957 (62.4)	166 (17.3)	791 (82.7)	786	566 (72.0)	Open dorm
Prison 2	39	1,247	1,236 (99.1)	0 (0.0)	1,236 (100)	1,157	893 (77.2)	Open dorm
Prison 3	21	1,070	997 (93.2)	0 (0.0)	997 (100)	992	551 (55.5)	Both
**California**								
Prison 1	27	3,175	257 (8.1)	39 (15.2)	218 (84.8)	217	34 (15.7)	Cells
Prison 2	18	3,739	441 (12.0)	6 (1.4)	435 (98.6)	433	8 (1.8)	Cells
Prison 3	2	2,325	54 (2.3)	0 (0.0)	54 (100)	54	23 (42.6)	Open dorm
Prison 4	41	3,419	2,153 (63.0)	15 (0.7)	2,138 (99.3)	2,128	371 (17.4)	Both
Prison 5	34	1,565	740 (47.3)	4 (0.5)	736 (99.5)	736	99 (13.5)	Cells
Prison 6	NA	3,327	92 (2.8)	0 (0.0)	92 (100)	92	0 (0.0)	Open dorm
**Colorado**								
Prison 1	28	2,340	2,296 (98.1)	1 (<0.01)	2,295 (99.9)	2,262	375 (16.6)	Cells
Prison 2	5	1,704	299 (17.5)	0 (0.0)	299 (100)	297	35 (11.8)	Cells
**Ohio**								
Prison 1	7	497	442 (88.9)	0 (0.0)	442 (100)	442	94 (21.3)	Both
Prison 2	12	2,521	2,510 (99.6)	0 (0.0)	2,510 (100)	2,510	2,179 (86.8)	Both
Prison 3	7	2,024	Unknown	Unknown	1,846	1,846	1,476 (80.0)	Both
**Orange County, California**								
Jail 1	34	3,167	1,002 (31.6)	0 (0.0)	1,002 (100)	1,002	374 (37.3)	Both
**Texas**								
Jail 1	27	7,800	1,070 (13.7)	0 (0.0)	1,070 (100)	1,070	519 (48.5)	Both
**Total**	**—**	**41,454**	**16,392 (39.5)**	**231 (1.6)**	**16,161 (98.6)**	**16,024**	**7,597 (47.4)**	**—**

* Mass testing was defined as offering SARS-CoV-2 testing by reverse transcription–polymerase chain reaction (RT-PCR) to all incarcerated or detained persons in at least one housing unit of a jail or prison, irrespective of presence or history of symptoms.

^†^ The first COVID-19 case in each facility was identified using a symptom-based approach.

^§^ The highest number of incarcerated or detained persons in the facility on a single day during the mass testing event.

^¶^ Some facilities offered SARS-CoV-2 testing to incarcerated or detained persons in all housing units. Others offered testing in selected housing units based on criteria including whether units had already identified cases, housed a large number of persons with underlying health conditions, or housed persons who were assigned to work details that required movements across the facility (e.g., food or laundry service).

** Open dorm units in these facilities housed from 63 to 216 persons in one space where they could interact freely. Cell-based units were comprised of locked cells housing from one to eight persons each.

^††^ The Federal Bureau of Prisons (BOP) has jurisdiction over federal prisons across the United States. The three BOP facilities with data presented here are located in three different states.

**FIGURE:**
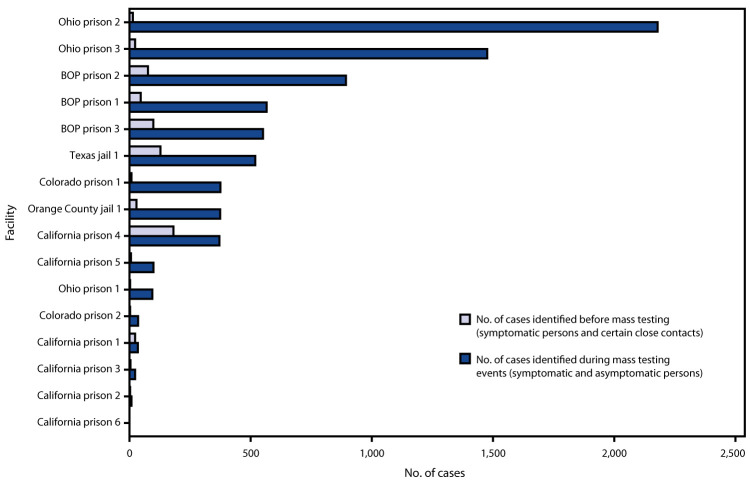
COVID-19 cases identified among incarcerated or detained persons through mass testing events (April–May) compared with symptom-based testing (January–April) in 16 prisons and jails — six jurisdictions, United States, 2020 **Abbreviations:** BOP = Federal Bureau of Prisons; COVID-19 = coronavirus disease 2019.

In addition to aggregate facility-level data, four of six jurisdictions provided mass testing data from 85 housing units within 12 of the 16 facilities. Forty-eight housing units were dormitory-based (open, communal spaces housing 63 to 216 persons in one room), and 37 were cell-based (with locked cells housing one to eight persons each). SARS-CoV-2 prevalence ranged from 1.8% to 45.0% (median = 14.6%) in cell-based units and 0% to 77.2% (median = 42.6%) in dormitory-based units.

In two federal prisons, all persons who had tested negative during mass testing events and had subsequently been quarantined as close contacts of persons testing positive were retested after 7 days. At retesting, 90 of 438 (20.5%) persons in BOP prison 2 and 84 of 314 (26.8%) in BOP prison 3 had positive test results.

Jurisdictions reported that mass testing results helped them construct medical isolation cohorts for persons testing positive and quarantine cohorts for their close contacts to prevent continued transmission. In some jurisdictions, results informed targeted testing strategies among asymptomatic persons in facilities where mass testing had not yet occurred (e.g., routine testing at intake, release, and before community-based appointments, and periodic testing of those assigned to work details requiring movement between different facility areas, such as food or laundry service). Jurisdictions reported that mass testing required large investments of staff member time and operational resources, and that the ability to rearrange housing based on test results was sometimes limited by space constraints. Jurisdictions stated that evidence-based recommendations about a potential role for less time- and resource-intensive testing (e.g., point-of-care antigen or antibody testing) and swabbing methods could help them expand testing in the future.

## Discussion

High SARS-CoV-2 prevalence detected during mass testing events in a convenience sample of correctional and detention facilities suggests that symptom-based testing underestimates the number of COVID-19 cases in these settings. Mass testing resulted in a median 12.1-fold increase in the number of known infections among incarcerated or detained persons in these facilities, which had previously used symptom-based testing strategies only.

Symptom-based testing cannot identify asymptomatic and presymptomatic persons,** who represent an estimated 40%–45% of infected persons across settings (*5*). Symptom-based testing might also be limited by hesitancy to report symptoms within correctional and detention environments because of fear of medical isolation and stigma (*6*). In the facilities included in this analysis, mass testing allowed administrators to medically isolate infected persons irrespective of symptoms and to quarantine their close contacts to reduce ongoing transmission. Testing refusal rates in these facilities of up to 17.3% highlight the need to communicate the importance of testing and address fear and stigma, with care to tailor messages to cultural and linguistic needs, and to develop strategies to reduce transmission risk from persons who decline testing.

High SARS-CoV-2 prevalence among persons quarantined and retested 7 days after an initial negative result indicates that curbing transmission in correctional and detention environments might require multiple testing rounds, coupled with other recommended prevention and control measures (*7*). Test-based release from quarantine could also be warranted. Serial testing among quarantined contacts of infected persons in a Louisiana correctional and detention facility found a 36% positivity rate 3 days after an initial negative result, indicating that a short retest interval could improve case identification (*8*).

This analysis can inform testing practices in correctional and detention facilities in at least three areas. First, testing staff members at regular intervals, regardless of symptoms, could become an important part of facilities’ COVID-19 prevention and mitigation plans, in collaboration with relevant stakeholders, including labor unions. In this study, more than half of the facilities identified their first case among staff members, consistent with previous CDC findings that staff members can introduce the virus into correctional and detention environments (*9*). Second, in descriptive analyses, the median prevalence of SARS-CoV-2 was nearly three times higher in dormitory-based housing units (42.6%) than in cell-based units (14.6%), suggesting that housing configuration might contribute to transmission. Further study is warranted to determine whether more frequent testing could reduce transmission in dormitory-based housing. Third, these mass testing events occurred 2–41 days after identification of the facilities’ first cases. Additional studies should examine whether timing of mass testing influences its effectiveness in facilitating outbreak containment. In a study involving five health department jurisdictions that conducted facility-wide testing in 88 nursing homes that had already identified at least one case, an estimated 1.3 additional cases were identified for each additional day between identification of the first case and completion of facility-wide testing, indicating that facility-wide testing early in an outbreak can be an effective mitigation strategy (*10*).

The findings in this report are subject to at least six limitations. First, these facilities represent a convenience sample and are not representative of all U.S. correctional and detention facilities. Second, because facilities’ decisions to conduct mass testing might be based on differing population characteristics, epidemiologic factors, and policy considerations, statistical significance testing was not performed. Third, the number of cases identified through mass testing might be higher in facilities where mass testing occurred closer to the peak of an outbreak (a factor that could not be determined with available data), or in facilities that tested a higher proportion of their population. Fourth, data regarding symptoms reported during mass testing were unavailable, preventing calculation of the percentage of persons with positive test results who were symptomatic. Fifth, cases among staff members identified before mass testing are likely underestimated because most facilities relied largely on self-reporting. Finally, it is uncertain whether the housing unit where a person with COVID-19 was tested was the location where exposure occurred.

Challenges in practicing physical distancing and other prevention strategies within correctional and detention facilities place persons in these settings, many of whom have chronic diseases, at high risk for SARS-CoV-2 exposure. This analysis demonstrates that mass testing irrespective of symptoms, combined with periodic retesting, can identify infections and support prevention of widespread transmission in correctional and detention environments. Further research is warranted to refine strategic testing approaches that individual facilities can implement, based on local needs and resources, to contribute to COVID-19 mitigation.

**Summary**

**What is already known about this topic?**

SARS-CoV-2 outbreaks in correctional and detention facilities are difficult to contain because of population-dense housing and limited space for medical isolation and quarantine. Testing in these settings has often been limited to symptomatic persons.

**What is added by this report?**

Mass testing in 16 U.S. prisons and jails found SARS-CoV-2 prevalence ranging from 0%–86.8%, a median 12.1-fold increase over the number of cases identified by earlier symptom-based testing alone. Median prevalence was three times higher in dormitory-based than in cell-based housing.

**What are the implications for public health practice?**

In correctional and detention facilities, broad-based SARS-CoV-2 testing provides a more accurate assessment of disease prevalence than does symptom-based testing and generates data that can potentially help control transmission.

## Footnotes

* Correctional facilities refer to state and federal prisons that incarcerate persons who have been tried for a crime, convicted, and sentenced for a duration of ≥1 year. Those convicted of federal crimes are incarcerated in federal prisons; those convicted of state crimes are incarcerated in state prisons. Detention facilities refer to jails or detention centers (including immigration detention centers) that temporarily detain persons awaiting trial, sentencing, or deportation, or those with a sentence of <1 year.

† Mass testing consisted of offering reverse transcription–polymerase chain reaction (RT-PCR) testing to all persons incarcerated or detained in at least one housing unit of a correctional or detention facility at a single point in time, irrespective of presence or history of symptoms.

§ Data elements collected included mass testing dates, facility census during testing, number of persons tested, number who declined, housing arrangements of persons tested, and test results.

¶ U.S. Department of Health and Human Services, Title 45 Code of Federal Regulations 46, Protection of Human Subjects.

** Presymptomatic persons are those who are infected with SARS-CoV-2 and do not have symptoms at the time of testing, but who develop symptoms later. Asymptomatic persons are those who are infected with SARS-CoV-2 but never develop symptoms. Both presymptomatic and asymptomatic persons can transmit the virus to others.
